# Hypoxia and the phenomenon of immune exclusion

**DOI:** 10.1186/s12967-020-02667-4

**Published:** 2021-01-06

**Authors:** Violena Pietrobon, Francesco M. Marincola

**Affiliations:** Refuge Biotechnologies, Inc., Menlo Park, CA USA

**Keywords:** Hypoxia, Immune exclusion, Tumor microenvironment, Physical barriers, Functional barriers, Dynamic barriers, Imaging

## Abstract

Over the last few years, cancer immunotherapy experienced tremendous developments and it is nowadays considered a promising strategy against many types of cancer. However, the exclusion of lymphocytes from the tumor nest is a common phenomenon that limits the efficiency of immunotherapy in solid tumors. Despite several mechanisms proposed during the years to explain the immune excluded phenotype, at present, there is no integrated understanding about the role played by different models of immune exclusion in human cancers. Hypoxia is a hallmark of most solid tumors and, being a multifaceted and complex condition, shapes in a unique way the tumor microenvironment, affecting gene transcription and chromatin remodeling. In this review, we speculate about an upstream role for hypoxia as a common biological determinant of immune exclusion in solid tumors. We also discuss the current state of ex vivo and in vivo imaging of hypoxic determinants in relation to T cell distribution that could mechanisms of immune exclusion and discover functional-morphological tumor features that could support clinical monitoring.

## Background

Over the last few years, cancer immunotherapy has experienced significant developments and is considered a promising therapeutic frontier against various types of cancer. Cell-based therapies could be encouraging strategies to eradicate cancer: chimeric antigen receptor T cells (CAR-T) and tumor infiltrating lymphocytes (TIL) are routinely expanded ex vivo and administered to patients. The most significant clinical responses have been obtained in haematological malignancies, using CAR-T lymphocytes engineered to recognize the CD19 antigen on neoplastic B cells [1–3]. Advanced clinical trials have also demonstrated promising results in various types of solid tumors [4–7]. Checkpoint blocking antibodies have been approved for the treatment of melanoma, head and neck squamous cell carcinoma, non-small lung cancer, urothelial bladder cancer and Hodgkin lymphoma [8–13].

Immunotherapy is particularly effective when applied for the treatment of cancers with an unstable genetic profile demarcated by high mutational burden. Previous studies have shown that neo-antigens are expressed by tumor cells and can be used as therapeutic targets [9, 14–16]. However, various challenges limit the efficacy of immunotherapy including a low mutational burden and low infiltration of CD8^+^ T lymphocytes into certain tumor areas [17, 18]. These obstacles limit the applicability of immunotherapies in a wide range of cancer types.

Spatial-functional orientation and density of CD8^+^ T cells within the tumor, have been shown to be directly associated with immunotherapy response and patient prognosis across many types of cancer [19–25]. Infiltration of immune cells can be detected by staining tissue samples with hematoxylin and eosin, or via immunostaining. Three topographies have currently been defined based on the distribution of T lymphocytes in the tumor: immune active (hot), immune desert (cold), and immune excluded. Hot tumors are enriched in CD8^+^ T cells while cold tumors may present with other immune populations or myeloid cells. Immune excluded tumors present cancer cell nests surrounded by T cells, which are incapable of penetrating inside [24, 26–29]. Although this classification is largely accepted, it is backed by little quantitative data about the relative frequencies of the three immune landscapes across cancers of different derivation.

The introduction of immunological biomarkers into tumor classification is currently under development. The Society for Immunotherapy of Cancer (SITC) Immunoscore Validation Project is a global and collaborative effort to validate a set of prognostic immune parameters centered predominantly on CD8^+^ T cell infiltration. These markers constitute the Immunoscore of a tumor as the objective calculation of T cells within a determined area that combines both intra-tumoral and per-tumoral infiltrates. This parameter could be used beyond its prognostic value also as a main predictor for the efficacy of cancer immunotherapies [30]. The Immunoscore project led to a multi-national immunohistochemistry study published in 2018, which reported the validation of a robust prognostic scoring system for the classification of colon cancer [26].

Transcriptional signatures, such as the immunologic constant of rejection (ICR) or the tumor inflammation signature (TIS), also demonstrated the importance of immune predictive biomarkers adding a functional dimension to the morphological parameters determining the Immunoscore [30–34]. A recent pan-cancer analysis highlighted the ICR signature as a predictive value in patients treated with anti-CTLA4 immune checkpoint inhibition [35]. However, further morphological and transcriptional studies are necessary to provide a deeper insight into the immune excluded phenotype.

Immune excluded tumors may occur as a result of several underlying mechanisms and present a considerable challenge for immunotherapy. Yet, they represent a unique model system, being different from homogeneously infiltrated tumors, as they present gradients of exclusion. Such gradients are peculiar within each tumor environment and possibly not present in silent tumors. In cold tumors instead, lack of chemo-attraction may constitute a predominant phenotype rather than the presence of barriers [36].

The variety of determinants potentially involved in immune exclusion suggested according to the literature, can be organized into three main groups: physical barriers, functional barriers and dynamic barriers. Physical or mechanical barriers include all mechanisms that prevent T cells from engaging with cancer cells, including structural components of the tumor microenvironment, vascular access and cancer cell coating [37]. In some cases, T lymphocytes may reach the tumor but metabolic barriers, soluble factors or tumor cell-intrinsic signaling impede their penetration and expansion into the tumor core. These are functional barriers. Finally, dynamic barriers may not be present in baseline conditions but are elicited only after the contact between T cells and cancer cells. An example for this kind of barrier is the inducible activation of PDL-1 in response to IFN-γ signaling [38–40].

At present, it is not clear if a predominant biology is responsible for most immune excluded cases, or if a synergy of random factors could better explain this complex phenomenon [18]. Moreover, a direct correlation has not been observed between tumor stages and the prevalence of a certain immune exclusion mechanism over another. In this review, we speculate on an upstream role played by hypoxia as a common biological determinant of immune exclusion. Hypoxia may be the initial triggering factor for most of the known mechanisms of immune exclusion. We also discuss the current state of ex vivo and in vivo imaging of hypoxic determinants and T cell distribution. Ex-vivo methods could facilitate the interpretation of spatial and temporal dynamics between these two parameters and elucidate if a specific sequential pattern is prevalent in most immune excluded tumors. In-vivo methods could be applicable in clinical settings, to monitor the efficacy of immunotherapy in hypoxic solid tumors.

## Molecular mechanisms involved in cell response to hypoxia

In normal physiological conditions, the partial oxygen pressure of a tissue is called physioxia. Depending on the tissue, it ranges from 8 to 100 mmHg (Table 1). For example, in venous blood the partial oxygen pressure is 30-40 mmHg while in arterial blood is 75–100 mmHg [41].

**Table 1:** Reference values of pO_2_ measurements in different human tissues

Human tissue	pO_2_ (mmHg)	References
Air	160	
Arterial blood	75–100	[434–436]
Venous blood	30–40	[436, 437]
Brain	39 ± 9	[438–442]
Lung	42.8	[443]
Liver	55.5 ± 21.3	[444–446]
Kidney	72 ± 20	[447]
Muscle	29.2 ± 1.8	[448–452]
Skin epidermis	8 ± 3.2	[452, 453]
Small bowel	61.2	[452, 454, 455]
Large bowel	57.6	[452, 454, 455]
Bone marrow	51.8 ± 14.5	[456]
Ovaries	88	[457]
Cornea	30.6 ± 3.1	[458]
Femur Bone	34 ± 1.6	[459]

Hypoxia is an environmental condition that occurs when the demand for oxygen exceeds supply and values of partial oxygen pressure in a tissue drop below physioxia. Normoxia is defined as the partial oxygen pressure normally present in the environment (21% of oxygen corresponding to 160 mmHg).

Hypoxia normally occurs during development: mammalian embryogenesis occurs at low oxygen concentrations (1–5%) and the gradient of oxygen itself functions as morphogen in many developmental systems [42, 43]. Hypoxia is also linked to many medical conditions including myocardial infarction, ischemic or hemorrhagic brain stroke, transient ischemic attack and late stages of some neurodegenerative diseases [43–47].

Hypoxic areas are present in most solid tumours, as cellular proliferation outgrows the blood supply, and often contain regions where the concentration of oxygen is lower than 5 mmHg [48–50]. Diffusion of oxygen to the tissues occurs at an average distance of 100–170 µm from the capillary itself, therefore solid tumors need to become angiogenic to grow beyond 1–2 mm in diameter [51–55]. Highly proliferative tumors accumulate a subpopulation of cancer cells distant from the blood vessels; even when tumor neo-angiogenesis occurs, it is structurally and functionally abnormal, and cancer cells experience chronic hypoxia. Acute hypoxia is transient and can be caused by the mechanical pressure exerted by fast growing tissues on existing blood vessels or by temporary fluctuations in blood perfusion in newly formed vessels. These phenomena lead to a metabolically heterogeneous tumor microenvironment [56–62].

These environmental conditions create a strong selective pressure on cells, favoring the growth of more aggressive tumor clones. Hypoxia is generally clinically associated with poor prognosis across multiple tumor types and is also one of the main causes of resistance to therapy. Hypoxic tumors are generally more aggressive, with increased metastatic potential and reduced apoptosis [59, 60, 63–66].

A compelling understanding of the molecular mechanisms involved in the hypoxic response has been cemented in the last 25 years. A family of heterodimeric transcription factors called Hypoxic Inducible Factors (HIFs) is responsible for the maintenance of cellular homeostasis during hypoxic conditions. They consist of an α (HIF-α) and a β (HIF-β or ARNT) subunit. There are three HIFα isoforms: HIF1-, HIF2- and HIF3-α. Therefore HIF-1, for example, is constituted by HIF-1α and HIF-1β. HIF proteins bind to Hypoxia Regulated Elements (HREs), canonical DNA sequences in the promoters or enhancers regions, activating the expression of more than 100 genes [67–69].

**Fig. 1:**
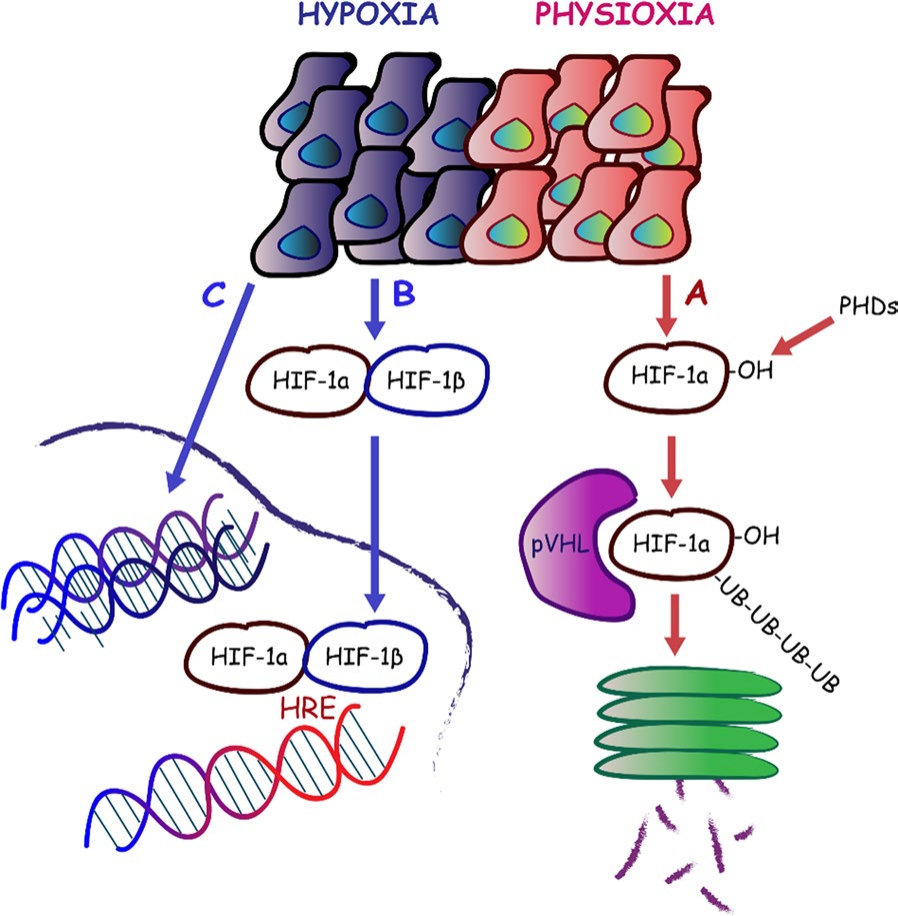
Mechanisms of HIF-1α protein stabilization in hypoxia and degradation in normoxia. **a** Under normal oxygen tension, HIF-α subunits are expressed, hydroxylated by a family of oxygen dependent prolyl hydroxylases (PHDs), recognised by the von-Hippel Lindau tumor suppressor (pVHL) which leads to HIF-α poly-ubiquitination and subsequent degradation by the 26S proteasome. **b** Under hypoxic conditions HIF-α is no longer hydroxylated but it dimerizes with the constitutively expressed HIF-β, enters the nucleus and binds to HREs to upregulate transcription of a group of hypoxic responsive genes. **c** Extensive modifications in chromatin structure, both HIF dependent and independent, also promote gene silencing

Under normal oxygen tension, HIF-α subunits are expressed but rapidly degraded resulting in minimal levels of detectable HIF proteins (Fig. 1a). Distinct proline sites, within the oxygen degradation domain (ODD) of HIF-α, are hydroxylated by a family of oxygen-dependent prolyl hydroxylases (PHDs). Hydroxylated HIF-α is recognised by the von-Hippel Lindau tumor suppressor (pVHL), leading to HIF-α poly-ubiquitination and subsequent degradation by the 26S proteasome [70–73]. Under hypoxic conditions HIF-α is no longer hydroxylated but it dimerizes with the constitutively expressed HIF-β (Fig. 1b). The heterodimer enters the nucleus and binds to HREs to upregulate transcription of a group of hypoxic responsive genes [68, 69, 74–78]. The HIF signaling pathway is activated not only by hypoxia but also by mutations that inactivate tumor suppressor genes such as *VHL.* Loss of function of the VHL protein causes an autosomal dominant hereditary disorder characterized by clear cell renal carcinoma, retinal, cerebellar and spinal hemangioblastoma and a multitude of visceral tumors. Somatic *VHL* mutations have also been implicated in sporadic renal carcinoma, accounting for approximately 80% of adult sporadic tumors [79–81]. The HIF pathway is also activated by increased activity of the phosphoinositol 3-kinase (PI3K) and mitogen-activated protein kinase (MAPK) signalling cascades [82–84].

PI3K and MAPK signalling cascades can regulate HIF-1α under normoxic conditions. The MAPK pathway is required for HIF-1α transactivation activity while PI3K can increase its mRNA translation through mechanisms dependent or independent on the mammalian target of rapamycin (mTOR) [85–88]. Another mechanism triggering the stabilization of HIF proteins is mediated by the intracellular increase in reactive oxygen species (ROS). ROS levels increase during acute and chronic hypoxia and are also a side effect of chemotherapy. This could represent one of the numerous mechanisms involved in tumor refractoriness to cytotoxic therapies [89].

HIF proteins activate the transcription of genes involved in stem cell maintenance [90], apoptosis, cell immortalization, epithelial to mesenchymal transition [91], genetic instability [92], erythropoiesis and angiogenesis [93], glycolysis [94], pH regulation [95], immune evasion [96], invasion and metastasis [97] and radiation resistance [43, 98]. The relationship between these transcriptional modifications and the immune excluded phenotype will be discussed in the next section.

HIF-1α and HIF-2α are structurally similar, with the exception of the transactivation domain. HIF-1α generally binds HREs close to gene promoters while HIF-2α targets transcriptional enhancers [68, 74, 99–102]. This could explain why, despite binding identical HRE sequences, they have both overlapping and unique target genes. The isoform specificity influencing the outcome of the transcriptional programs has been investigated in several studies and found to vary depending on cell type, genetic background, severity and duration of hypoxia [103–107]. While HIF-1 plays a major role in glycolytic gene regulation, HIF-2 is mainly involved in pluripotent stem cell maintenance and angiogenesis, enhancing the pro-tumorigenic phenotype [108–110]. HIF-1α is mainly expressed during acute hypoxia (in the first 24 hours) in all tissues, while HIF-2α is stabilized later and its expression is limited to specific tissues [110–112]. Although the expression of HIF-3α is detectable in a variety of human cancer cell lines, it has been less investigated. HIF-3α lacks a transactivation domain, suggesting that this form possesses a suppressive effect toward the other HIF isoforms [113–116].

Interestingly, under hypoxic conditions, there are also substantial HIF-independent changes in global gene transcription. Vast transcriptional repression forms a significant component of the hypoxic response which is mediated, in part, by at least ten different transcriptional repressors [117, 118]. Extensive modifications in chromatin structure, both HIF dependent and independent, promote gene silencing (Fig. 1c). High-throughput RNA-seq of human embryonic kidney cells revealed 851 and 1013 genes induced and repressed in hypoxia, respectively [117]. Transcriptomic studies in kidneys from ischemic mice revealed that 642 genes were induced, while 577 were repressed [118].

Downregulated genes include those coding proteins associated with oxidative phosphorylation, transcription, translation and mRNA processing, intercellular junctions and DNA repair pathways. These latter include BRCA1, BRCA2, RAD51 genes, and genes involved in mismatch repair and nucleotide excision repair [119–121]. Their transcriptional and translational repression leads to moderate hypoxia-driven genomic instability [122–125]. DNA replication stress is also a HIF-independent phenomenon triggered by hypoxia and it is caused by a decreased activity of oxygen-dependent replication enzymes [126]. During transient episodes of re-oxygenation, hypoxic cells may undergo further DNA damage as a result of a burst of free radicals [127, 128]. Studies published so far demonstrate how diverse and important the changes associated with the transcriptional landscape in hypoxia are, and how the metabolic traits of cells experiencing hypoxia are profoundly impacted.

## Hypoxia as an upstream determinant in immune excluded phenotypes

Hypoxia occurs during cancer development, primarily because cellular proliferation outgrows its blood supply. Hypoxia response does not translate in a random activation of cellular pathways which are responsible to keep homeostasis, but it is responsible for an organized and evolutionary-established progression of events [43].

Despite HIF-1 and HIF-2 sharing a pool of target genes, there are other genes specifically upregulated by HIF-1 or HIF-2. As mentioned before, other differences between the two proteins include their kinetics of expression; stabilization of HIF-1 occurs in the first phases of hypoxia (acute) leading to upregulation of genes mainly involved in cell metabolism and angiogenesis, while HIF-2 is generally stabilized later (chronic). Their stabilization is also tissue dependent [101–105].

This background provides some rationale for the importance of having a spatial and temporal topographic map of the correlation between hypoxic phenotypic expressions and immune exclusion in different types of cancer.

### Mechanical barriers

**Fig. 2:**
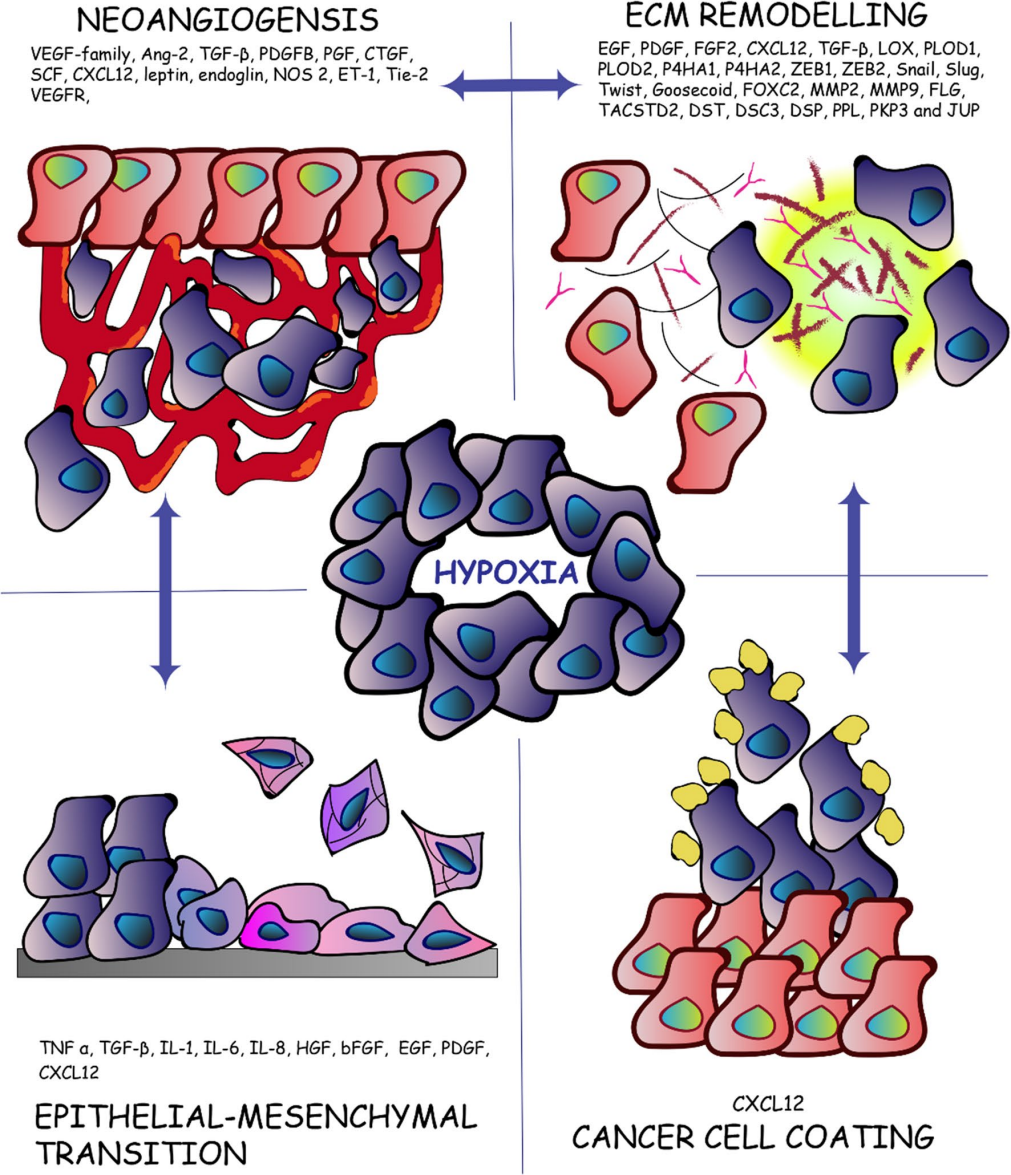
Hypoxia-induced mechanical barriers. Mechanical barriers represent a category of determinants of immune exclusion where a lack of interaction between T cells and cancer cells is due to physical impediments. Hypoxia induced-mechanical barriers include neoangiogenesis (vascular accessibility), ECM remodeling (stromal fibrosis, collagen remodelling and crosslinking), EMT and cancer cell coating

Mechanical barriers represent a category of determinants of immune exclusion, where lack of interaction between T cells and cancer cells occurs due to physical impediments. These impediments include vascular accessibility, stromal fibrosis and cancer cell coating (Fig. 2).

Through upregulation of HIF-induced genes, cancer cells produce pro-angiogenic factors, affecting the equilibrium between pro- and anti- angiogenic factors in the tumor microenvironment (TME). The newly formed vessels are often structurally and morphologically aberrant: leaky, tortuous, compressed or dilated. The consequence is the development of a pro-tumorigenic environment with a spatial-temporal heterogeneity in blood flow. Blood vessels anomalies lead to increased blood viscosity, reduced flow, enhanced hypoxia, acidosis, high interstitial fluid pressure and immunosuppressive TME [54, 129–132]. This plethora of conditions play a pivotal role in affecting T cell delivery and fitness into the tumor core and thus the clinical response to immunotherapy [133–135].

HIF-1 increases angiogenesis and vascular permeability by upregulating a variety of angiogenetic factors including those belonging to the VEGF-family, angiopoietin-2 (Ang-2), transforming growth factor beta (TGF-β), platelet-derived growth factor B (PDGFB), placental growth factor (PGF), connective tissue growth factor (CTGF), stem cell factor (SCF), stromal cell-derived factor 1 (CXCL12), leptin, endoglin, nitric oxide synthase 2, haemoxygenase-1 and endothelin-1 (ET-1) [136–142]. It has been shown that HIF-2 upregulates VEGF receptor-2 [143] and the endothelial receptor tyrosin kinase Tie-2 [69].

ET-1 is a vascular regulator peptide, which promotes angiogenesis directly and indirectly. However, HIF-1 binding to ET-1 promoter is not sufficient and transcriptional activation requires binding of activator protein-1 (AP-1), GATA-2, and CAAT-binding factor (NF-1) [144]. ET-1 acts through two G-protein coupled receptors: Endothelin A and B receptors. It has been shown that ET-1 promotes neo-angiogenesis and increases vascular permeability through Endothelin B receptor activation [145]. The result is the increased survival and proliferation of endothelial cells and the upregulation of VEGF factors. Evidence shows a positive correlation between ET-1 and VEGF expression in a variety of different tumors [144, 146, 147]. Transcriptional profiling of micro-dissected tumor endothelial cells revealed that overexpression of the Endothelin B receptor is associated with the absence of tumor-infiltrating lymphocytes and worse prognosis in patients with ovarian cancer [148].

VEGF is also produced by endothelial cells, fibroblasts and inflammatory cells and it has been implicated in fibrosis as well. VEGF induces the reorganization of the extra-cellular matrix (ECM) through activation of stromal cells and induction of fibronectin and collagen type-I [149]. Angiogenic factors also contribute to fibrosis, attracting fibroblasts or activating resident fibroblasts. This normally occurs during wound healing, when angiogenesis and ECM deposition happen concomitantly [150].

A variety of cytokines secreted by cancer cells, and other stroma cells, is responsible for fibroblast recruitment and activation: epidermal growth factor (EGF), platelet-derived growth factor (PDGF), fibroblast growth factor 2 (FGF2), CXCL12 and TGF-β are key regulators of fibroblast recruitment and activation [151–158]. In hypoxia, HIF proteins and TGF-β are reciprocally induced, in a positive feedback loop. TGF-β is a main player in the activation of recruited and resident myofibroblasts and fibroblasts in the primary tumors, transforming them in cancer-associated fibroblasts (CAFs) [153]. CAFs are morphologically different from normal fibroblasts and posses increased proliferative and migratory potential. CAFs are responsible for the production of fibrous material and the secretion of cytokines, playing multiple roles in the TME. In addition, they contribute to angiogenesis, ECM remodeling, metastasis, metabolic reprogramming and immune regulation. Therefore, CAFs actively contribute to the development of mechanical and functional barriers [151, 159–162].

Studies on CXCL12, which is mainly secreted by CAFs, demonstrated its dual role as functional and physical barrier. CXCL12 plays a role in tumor growth, angiogenesis, immune suppression and invasion [142, 163]. However, recent evidence showed how this cytokine could have a role as a physical barrier in pancreatic ductal adenocarcinoma (PDA). PDA-bearing mice display reduced response to immunological checkpoint antagonists because CXCL12 could coat cancer cells and shield them from T cells [164, 165].

CXCL12 is also one of the chemokines involved in epithelial-mesenchymal transition (EMT) in different types of cancer [166, 167]. EMT is a process through which epithelial cells gain migratory and invasive properties, detaching from the basement membrane and acquiring properties similar to mesenchymal stem cells. This process is driven by inflammatory cytokines and it is also responsible for the development of mechanical barriers [37, 168]. Hypoxia-induced cytokines, which trigger EMT, also include tumor necrosis factor α (TNF-α), TGF-β, interleukin 1 (IL-1), interleukin-6 (IL-6) and interleukin-8 (IL-8). Cytokines are secreted by cancer cells, myeloid cells and mesenchymal cells. In many carcinomas, together with TGF-β and interleukins signaling, hepatocyte growth factor (HGF), basic fibroblast growth factor (bFGF), epidermal growth factor (EGF) and PDGF also play a role in the induction of transcription factors responsible for EMT progression [169].

It is common knowledge that tumor stroma presents increased hypoxia-dependent stiffness due to the increased secretion of fibrous material and collagen-modifying enzymes. Collagens constitute up to 90% of the ECM and the most prevalent ECM alteration in tumors is, indeed, an increase in collagen deposition. It has been demonstrated that in breast tumors the stroma can be up to ten times stiffer than in normal tissue, with an increase in cross-linked collagen fibers and other ECM components [170–174]. Transcription factors responsible for the synthesis of ECM components and remodeling enzymes are zinc finger E-box binding homeobox 1 and 2 (ZEB1, ZEB2) proteins, Snail, Slug, Twist, Goosecoid, and FOXC2. Collagen crosslinking is initiated in the extracellular compartment by a family of secreted enzymes called LOX. In different cancer cell lines, HIF proteins upregulate different members of the LOX family and all of them have been shown to be involved in tumor fibrosis [175–177].

Another mechanism of hypoxic-induced fibrosis is the upregulation of PLOD1, PLOD2 and P4HA1, P4HA2 genes which encode for proteins that are necessary for the biogenesis of collagen. This mechanism also includes increased production of procollagen lysyl and prolyl hydroxylases, respectively. These genes are not exclusively induced in cancer cells, but also in fibroblasts, chondrocytes and endothelial cells. Increased expression of PLOD2 in breast cancer and sarcomas has been associated with tumor stiffness and increased metastatic potential [178, 179]. Depletion of HIF-1, but not HIF-2, inhibited collagen deposition in vitro, and decreased tissue stiffness in orthotopic tumors [180–183]. Collagen degradation is also a part of the TME remodeling, and matrix metalloproteinases (MMPs) are responsible for this process. HIF-1 is associated with transcriptional upregulation of MMP2 and MMP9 in vitro.

Overall HIF factors reshape the transcriptional landscape of ECM, resulting in the increase of fibrillar collagens and degradation of the basement membrane. ECM remodeling leads to increased stiffness which influences the bioavailability of signaling molecules and the accessibility of T cells. In correlation with these findings, is the reduced infiltration of CD8^+^ T cells, which was observed in breast tumors with high collagen-density. Transcriptome analysis of 3D-cultured T cells on high density matrix showed downregulation of cytotoxic markers suggesting reduced engagement with antigen-bearing cancer cells [184].

A recent publication by Salerno et al., revealed that human melanoma and ovarian cancers lacking a Th1-polarized immune signature display upregulation of genes encoding for mechanical barrier function in the skin. Filaggrin (FLG), tumor-associated calcium signal transducer 2 (TACSTD2) and six desmosomal proteins (DST, DSC3, DSP, PPL, PKP3, and JUP) were upregulated, but the most upregulated one was *Flg* [37]. Interestingly, it has been previously demonstrated that expression of *Flg* is upregulated in a HIF-1- and HIF-2-dependent manner [185].

### Functional barriers

**Fig. 3:**
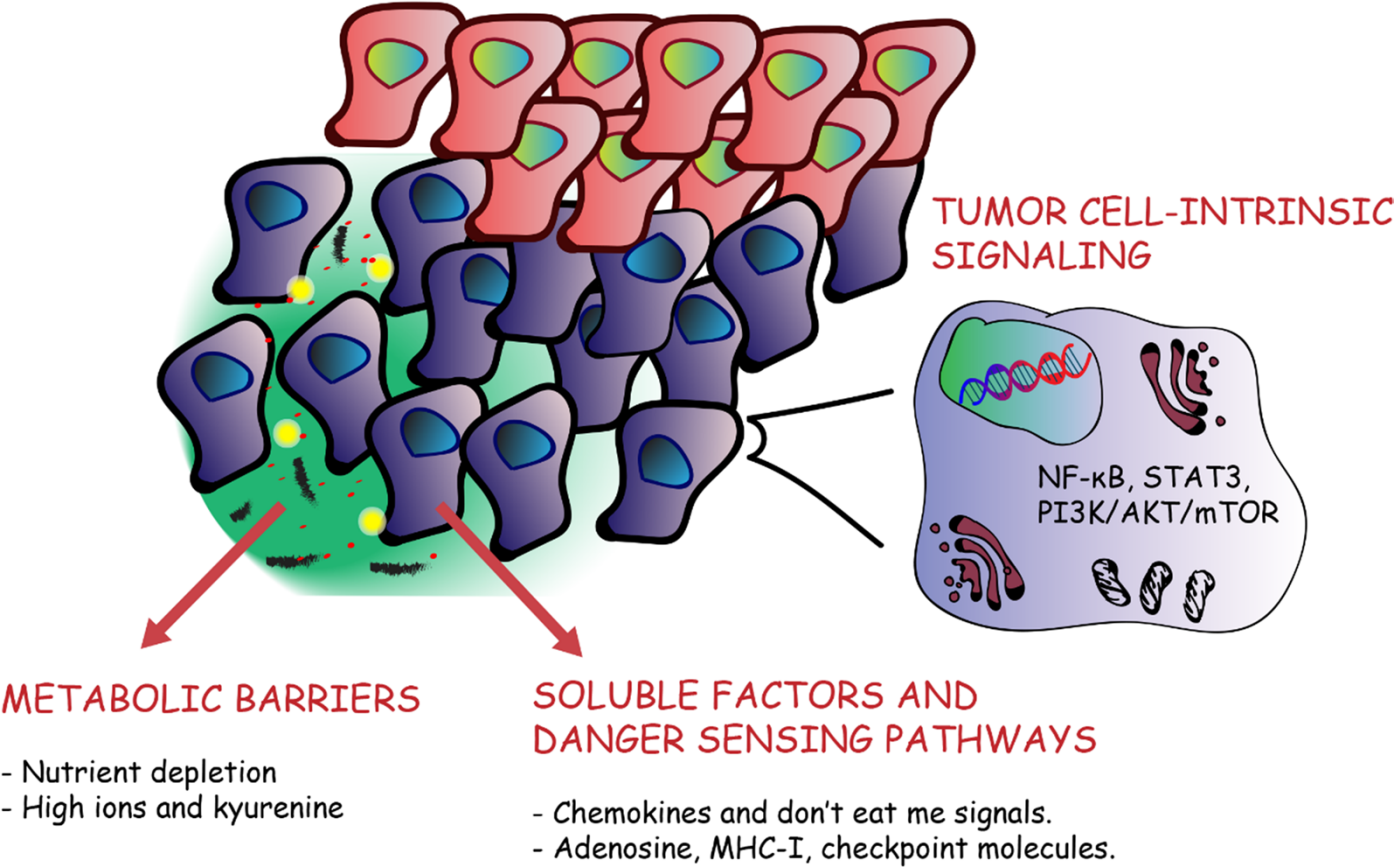
Hypoxia-induced functional barriers. Functional barriers represent a class of impediments in which T cells are in close proximity with cancer cells, but their activity is impaired. Hypoxia-driven functional barriers include metabolic barriers, secreted soluble factors, danger sensing pathways and/or cell-intrinsic signaling

Functional barriers represent a class of impediments in which T cells engage with cancer cells but their activity is impaired. Mechanisms such as metabolic barriers, soluble factors, danger sensing pathways and/or cell-intrinsic signaling affect T cell penetration and expansion in the tumor nests (Fig. 3).

#### Metabolic barriers

Nutrient depletion of the TME by cancer cells represents a classic example of a mechanism leading to metabolically-determined functional barriers. Moreover, a shift of cellular metabolism from oxidative to glycolytic occurs almost universally in cancers and it is known as the Warburg effect. Although this phenomenon has been described initially in cancer cells, it has also been observed in normal, but rapidly proliferating cells [186, 187]. Interestingly, cancer cells generally exhibit higher glucose metabolism, even when oxygen levels are comprised in a physiological range [188].

In response to hypoxia, HIF-1 orchestrates an evolutionary conserved response regulating oxidative metabolism. The transition from oxidative to glycolytic metabolism occurs when oxygen levels become limiting for mitochondrial ATP production. HIF-1 upregulates the enzymes glucose transporters (GLUTs) 1–3 to maintain cellular ATP pools [189–191]. GLUTs overexpression in solid tumors is correlated with poor prognosis and found in a variety of malignant neoplasms [192]. HIF-1 is also responsible for the upregulation of enzymes involved in glycolysis, such as pyruvate dehydrogenase kinase 1 (PDK1) which phosphorylates and inactivates the pyruvate dehydrogenase. Other proteins upregulated in hypoxia are lactic dehydrogenase A (LDHA) and pyruvate kinase M2 subtype (PKM2). Increased glycolysis leads to the production of pyruvate, which is subsequently converted into lactate [193].

Lactate, responsible of lowering intracellular pH, is exported to the ECM via mono-carboxylate transporters (MCTs are upregulated), leading to the acidification of the ECM. In the TME, lactate can reach concentrations of 20-30 mM, compared to 3 mM in normal tissues [194, 195]. High lactate concentration decreases T and NK cell function and survival and extracellular acidification is one of the causes leading to T cell immune response impairment [196, 197]. Other pH regulating enzymes, that are induced in hypoxic conditions, include carbonic anhydrases (CAs), Na+/H+ exchanger (NHE1), bicarbonate transporters (SLC4A4) and indoleamine 2,3 dioxygenase (IDO). These enzymes are responsible for the acidification and tryptophan depletion of the TME, which suppress T cell activity [95, 198–201].

In addition to glucose, essential amino acids can also be deprived from the TME. Myeloid-derived suppressor cells produce arginase [202, 203], while CAFs can process tryptophan leading to the downstream production of kyurenine, a suppressive metabolite [204, 205]. However, controversial evidence has been reported regarding tryptophan metabolism. Schmidt et al., showed in different human cell lines that hypoxia leads to a decrease in IDO expression which causes reduced production of kynurenine [206]. Recent studies have demonstrated how an alternative pathway via tryptophan-2,3-dioxygenase (TDO2) is also responsible for suppressing antitumor immune response in a variety of cancers [207]. Another study has shown that HIF-1 inhibits the expression of TDO2 in glioblastoma and hypoxic conditions increase T cell proliferation [208].

A further determinant which can act as a functional barrier to T cell activity is the lack of extracellular glutamine [209, 210]. T cells and cancer cells proliferation is associated with increased consumption of glutamine. Glutaminase 1 (GLS1) is a mitochondrial enzyme which hydrolyzes glutamine into glutamate and provides carbon and nitrogen crucial for anabolic metabolism and thus for rapid cell proliferation. It has been demonstrated that downregulation of glutamine and leucine metabolism inhibits the differentiation of TH1 and TH17 effector lymphocytes while maintaining T reg differentiation [211].

Xiang et al., demonstrated that HIF-1 binds directly to GLS1 promoter, inducing this gene in hypoxic colorectal cancer cells and increasing glutamine conversion to glutamate [212]. Hypoxia-induced transcriptional changes lead to a decrease in the extracellular concentration of glutamine, which plays a role in T cell regulation and activation [211, 213] .

Chronic hypoxia leads also to downregulation of Kv1.3, which is a classical Shaker-type potassium channel with six transmembrane segments in a variety of different cell lines [214]. Hypoxia has been reported to promote both, acute and long-term inhibition of Kv1.3 in T lymphocytes [215, 216]. Therefore, it is not unusual to detect elevated extracellular potassium concentrations in solid tumors. An ionic misbalance may lead to a state of functional caloric restriction in T cells triggering a starvation response, with T cells retaining stemness.

T cells rely on glycolysis to proliferate and secrete cytokines and cytotoxic mediators. Therefore, low pH, low glucose and reduced amino acid presence lead to T lymphocytes exhaustion. Exhausted T cells undergo metabolic and functional impairments, display mitochondrial dysfunction, high levels of co-inhibitory receptors (PD-1, CTLA-4, Tim-3, lymphocyte activation gene-3) and low proliferative capacity.

#### Soluble factors and danger sensing pathways

Compelling reviews have recently been published, summarizing the contribution of hypoxia in cytokine mediated immunosuppression [217, 218] and it is not the aim of this review to describe in detail such phenomenon. Briefly, hypoxia is a major suppressor of the immune system, altering the expression of cytokines, recruiting suppressive cell populations and expressing co-inhibitory ligands.

HIF-1 induces the secretion of a variety of chemokines responsible for myeloid cell recruitment, such as CCL5 and CXCL12 by cancer cells [219, 220]. Moreover, the expression of CXCR4 by myeloid and tumor cells is also regulated by HIF proteins [221]. VEGF, Sema3A, CCL28, endothelin 1 and 2 secretion is induced in hypoxia and they act as chemo-attractants for monocytes, macrophages and T regulatory cells (T regs) [222–226]. TGF-β is a pivotal player in the immune surveillance mechanism, acting directly and indirectly to restrict and modulate T cell trafficking [227]. As mentioned in the previous section, TGF-β secretion is induced by HIF-1 creating a positive feedback. Indeed, in hypoxia, immune suppressive populations such as T regs, tumor-associated macrophages (TAMs) and myeloid-derived suppressor cells (MDSCs) prominently infiltrate the tumor site, reducing the access of cytotoxic T cells and natural killer cells. Hypoxia also enhances the synthesis of CD39 and CD73 enzymes, which are important factors in the immunosuppressive mechanism involving adenosine production in the TME [228–230]. Adenosine is produced by hydrolysis of tumor cell derived ATP and ADP, and released in the TME through membrane channels, cell death or granular components. Adenosine receptors on T cells are transcriptionally induced by HIF-1 and HIF-2 in hypoxia, leading to the accumulation of immunosuppressive intracellular cAMP [64, 231].

Siemens et al., reported that hypoxia induces downregulation of different molecules, necessary for effector immune cells recognition. Therefore, tumor cells render themselves virtually invisible to cytotoxic T lymphocytes [232]. *In vitro*, hypoxic tumors can downregulate expression of major histocompatibility class-I (MHC-I) molecules reducing the recognition by cytotoxic T cells [233]. In vivo studies demonstrated that hypoxia leads to the inhibition of IFN-γ–dependent MHC class I upregulation. Such phenomenon occurs concomitantly with CXCL9 and CXCL10 transcriptional downregulation and it is reversible upon re-oxygenation [234]. In hypoxia, another functional barrier is created by the upregulation of immune checkpoint molecules (CTLA-4, PD-L1, HLA-G). It has been demonstrated that the PTEN/PI3K pathway through HIF-1 is responsible for this phenotype, in several different mouse and human tumor cell lines [218, 235–240].

Finally, TAM receptor kinases and “Don’t eat-me” signals (CD47/signal regulatory protein (SIRP)-α axis) constitute another example of functional barriers. In triple-negative breast cancer cells and in pancreatic adenocarcinoma HIF-1 induces the expression of CD47, leading to escape from immune surveillance [241–243]. Interaction of CD47 with SIRPα causes the block of pro-phagocytic signals and tumor cells escape from macrophages and MDSC mediated phagocytosis. Inhibiting phagocytosis has an anti-inflammatory role, through the activation of innate immune effector cells [244, 245].

TAM receptors such as Tyro3, Axl and Mertk are a family of transmembrane receptor tyrosine kinases which coordinate immune cell activity by promoting the phagocytosis of apoptotic cells. TAM receptors bind to eat-me signal phosphatidylserine displayed on apoptotic cell membranes, with the help of bridging ligands Gas6 and ProteinS. TAM receptors share overlapping functions in tumorigenesis and suppression of anti-tumor immune response [246, 247]. Several studies showed that TAM receptors are involved in the hypoxic response. For example, HIF proteins transcriptionally upregulate Axl in clear cell renal carcinoma [248]. Hypoxia is also responsible for stabilizing GAS6/Axl signaling in metastatic prostate cancer [249]. Tyro3 play a role in promoting survival of neurons and brain endothelial cells exposed to hypoxia and other stressors [250–253]. Activation of these tyrosine kinases is generally coupled to the downstream activation of the PI3K/AKT pathway or the Janus kinase (JAK)-signal transducer and activator of transcription (STAT) pathways. Differential activation of these two signaling cascades may lead to the regulation of distinct TAM-associated functions [246, 254–256]. Finally, Mertk knockout mice present with increased CD8^+^ T cell infiltration and higher levels of inflammatory cytokines (IL-12 and IL-6) levels. Change in cytokine expression may be responsible for the polarization of the TME toward an immune responsive type [247, 257].

#### Tumor cell-intrinsic signaling

A variety of oncogenic tumor-intrinsic pathways could be related to the immune excluded phenotype. A few examples will be given for those that present higher proofs of correlation with hypoxia and the excluded phenotype. Hypoxia induces NF-κB activation in different tissues, and it seems that a positive regulatory loop between HIF-1 and NF-κB exists, which may be cell line dependent [258–262]. NF-κB is a transcription regulator and its constitutive activation is considered as a hallmark of tumors. NF-κB mediates EMT, in cooperation with TGF-β, and shapes cancer stem cells (CSCs) features [263, 264]. Generally, the NF-κB signaling pathway promotes the secretion of cytokines that regulate immune response (e.g., TNFα, IL-1, IL-6, and IL-8) as well as adhesion molecules responsible for the regulation of leukocyte recruitment [265].

NF-κB inhibitor DHMEQ reversed the immunosuppression of human dendritic cells and macrophages cultured in the supernatant of epithelial ovarian cancer cells [266]. Evidence regarding NF-kB in a cancer context are controversial, as it has been shown that NF-κB can trigger production of chemokines necessary to recruit activated T lymphocytes to the tumor site [267, 268]. The biological significance of NF-kB signaling could be dependent on the cellular composition of cancers.

STAT3 is a transcriptional factor which cooperates with HIF-1 in activating hypoxic target genes. This has been shown in a variety of cell lines [269, 270]. The STAT3 signaling pathway is associated with tumor growth and reduction of T cell infiltration [271]. Blocking the STAT-3 pathway in tumor cells results in tumor-specific T cell responses due to the increase in the expression of pro-inflammatory cytokines and chemokines [272]. STAT3-HIF-1 signaling also enhances EMT in esophageal cancer and promotes immunosuppression through M2 polarization in glioblastoma [273, 274]. Moreover, Ihara et al., reported increased immune response in the absence of STAT3 dependent signaling associated with an increase in CCL5 and CXCL10 [275].

Similarly, for STAT3, β-catenin can interact with HIF proteins promoting adaptation to hypoxia. A variety of non-inflamed tumors show WNT/β-catenin signaling pathway activation and in vivo experiments in melanomas proved that activation of the β-catenin signaling can dominantly exclude immune cell activation [276, 277]. In addition, β-catenin-overexpressed by melanomas inhibits the production of IFN-γ by melanoma-specific cytotoxic lymphocytes, in an IL-10-independent manner [278] .

The PI3K/AKT/mTOR pathway is found to be active in most tumors. PI3K activation can be due to increased expression of growth factor receptors (such as EGFR), RAS mutation or loss of PTEN [279–281]. Loss of PTEN function increases PD-L1 expression and consequent immune resistance, in gliomas [282]. Moreover, deletion of PTEN in mouse models of sarcomas and prostate cancer demonstrated that it plays a role in tumor progression causing the infiltration of myeloid-derived hematopoietic cells [283–285]. However, constitutive PI3K activation due to loss of PTEN in triple-negative breast cancer is associated with the infiltration of T lymphocytes in the TME [286].

Interestingly, the HIF-1 protein level can be regulated by activation of the EGFR/PI3K/AKT/mTOR pathway, leading to a hypoxia-mimicking phenotype [287]. Activation of the PI3K/AKT/mTOR signaling cascade triggers, via HIF-1 dependent and independent mechanisms, the secretion of VEGF proteins. Furthermore activation of PI3K leads to secretion of TNF, IL-6, CSF-1, VEGF-A, and IL-8 contributing to immunosuppressive cell recruitment and accumulation, such as TAMs [288–290].

PI3K/AKT signaling is vital for cell and survival but it seems to be also involved in cancer cell stemness maintenance [291]. Cancer stem cells normally represent a small population in the tumor, and they are mainly associated with the immune silent phenotype, in the hypoxic niche. HIFs protein stabilization is responsible for the adoption of stem cell properties, including multipotency and self renewal [292]. During cancer progression, stemness of cancer cells is maintained by hypoxia through different mechanisms, including enhancement of EMT and the transcriptional induction of stemness related genes (Oct4, POU5F1, Sox2, Nanog, BMI1, Myc and KLF4) [293–295]. Signaling cascades responsible to maintain stem-like features are also upregulated: BMP, Notch, WNT, JAK-STAT, and Sonic hedgehog (Shh), TGF-β, SIRT1 and IL-6/STAT3. VEGF has also been found to play a role in stemness maintenance and it is produced by cancer stem cells under hypoxia [291, 295, 296]. HIF-1 and HIF-2 are differently expressed in CSCs but they are both required for their stemness, proliferation and survival, as well as upregulation of different subset of genes [297, 298]. The presence of HIF-2 has more frequently been detected in CSCs and, for example, its ability to regulate Oct4 is not shared with HIF-1 [299].

Finally, reduced expression levels of the immune signature have been found in tumor samples with elevated arm and whole-chromosomes somatic copy number alterations (SCNAs). Davoli et al. (2017) performed a bioinformatic analysis on 5255 tumor and normal samples representing 12 cancer types and found that reduced expression of markers for cytotoxic immune cell infiltrates was correlated with high levels of SCNAs [300]. Recently, further bioinformatic studies in different types of cancers confirmed that elevated levels of SCNAs are correlated with a decreased immune signature or an increase in pathways referring to immune suppression [301–303]. As mentioned in the previous chapter, hypoxia is responsible for a moderate genome instability, due to the transcriptional and translational repression of genes involved in DNA damage repair pathways. Replication stress is also a common feature of hypoxic cells [119–128]. Therefore, copy number variations due to an increase in genome instability might be a further mechanism through which hypoxia could lead to an immune excluded phenotype.

### Techniques to map hypoxic areas and immune infiltration

Hypoxia is a hallmark of most solid tumors and is associated with the malignant characteristics of cancers. It is also considered one of the many impediments to effective cancer therapy and is, therefore, correlated with poor prognosis. Both chronic and acute hypoxia lead to a variegated TME, exposing cells to different partial oxygen pressures and probably to gradients of immune infiltrations.

**Fig. 4:**
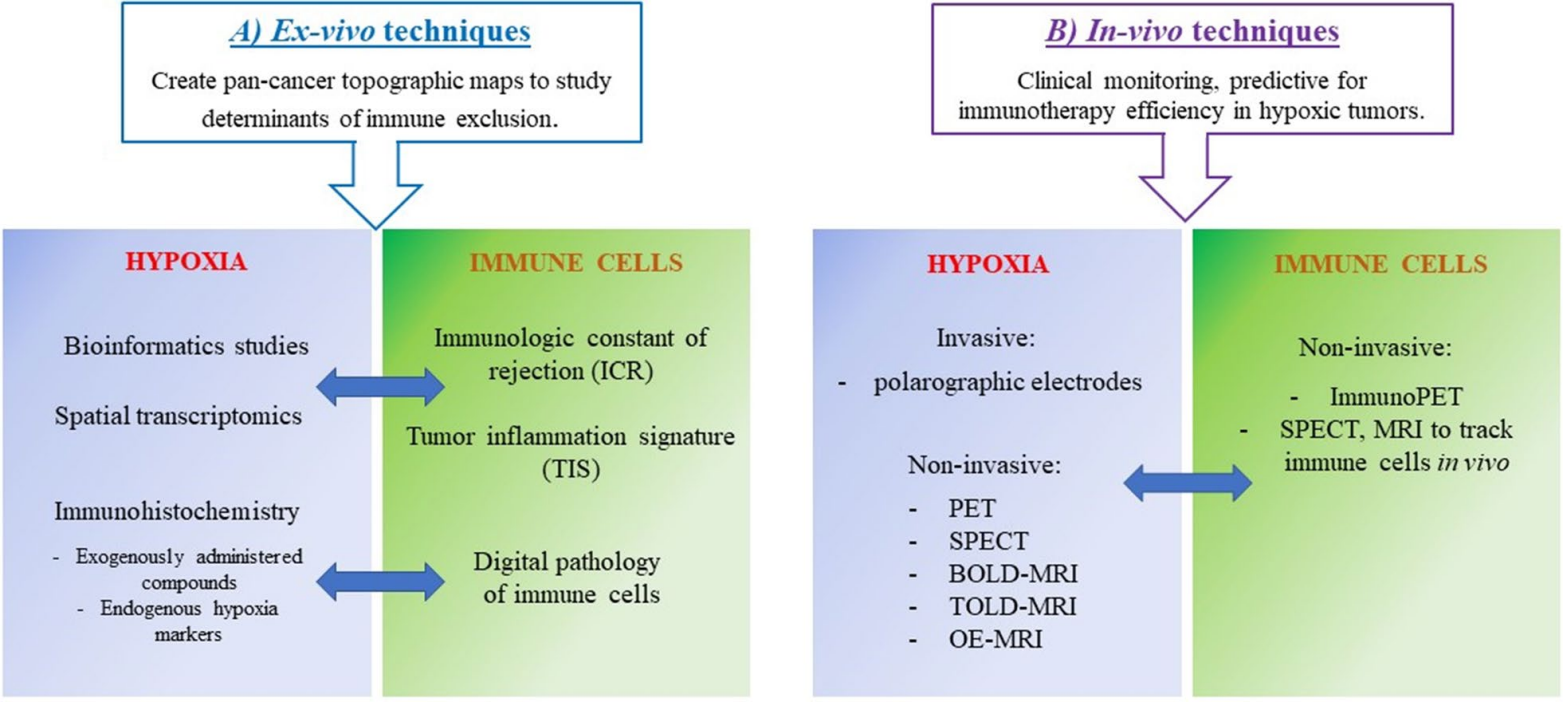
Scheme of ex vivo and in vivo techniques to detect hypoxia and immune cells. **a** Ex-vivo techniques could be useful to create topographic maps of hypoxic-induced physical and functional barriers in immune excluded tumor areas, providing mechanistic highlights into the organization of immune exclusion determinants. Bioinformatic and spatial transcriptomics could investigate hypoxic transcriptional profiles in correlation with ICR or TIS. Immunohistochemistry of exogenous compounds or HIF-induced proteins coupled to digital pathology of immune cells could provide functional and morphological insights. **b** In-vivo techniques to detect gradients of hypoxia and immune infiltration may be useful to monitor immunotherapy efficiency in hypoxic tumors (clinical setting). To detect hypoxia and immune cells in patients, non-invasive methodologies are preferred

In order to have a mechanistic understanding on how functional and physical barriers correlate with morphological parameters in human cancer, different ex vivo tissue analysis techniques can be performed (Fig. 4a). These techniques could allow the creation of an accurate pan-cancer topographic map of hypoxic conditions in immune excluded areas and study the putative role of hypoxia as a possible upstream determinant for most immune excluded phenotypes.

A variety of bioinformatics studies in solid tumors revealed a correlation between high-hypoxic signatures and low immune infiltration. However, the relationship between hypoxia and immune exclusion has been investigated in few cancer types and a consensus on this aspect has not been reached. Bioinformatic studies did not allow to draw generalizable conclusions, highlighting the need for more comprehensive analyses of large bulk transcriptomic cohorts [304–307].

These analyses rely on data from public cancer databases and do not provide any spatial or temporal resolution but rather a ballpark assessment of T cell functional status in bulk tumor samples. In these studies, hypoxia signature is represented by a set of genes that are found to be induced, and a variety of different predictors of hypoxia have been reported during the years. Heterogeneity is an important feature in cancer, therefore such signatures are likely to be tissue-specific and, probably, cancer stage-specific. Efforts have been made by several groups to create a universal hypoxic profile relevant to various cancer types [308–310].

Spatial transcriptional profiling of hypoxia-induced genes coupled with immune signatures (such as ICR or TIS) [30–34], could be an interesting option to characterize hypoxic-dependent phenotypes related to immune exclusion. Hypoxia could be transcriptionally monitored through different marker mRNAs such as those for HIF-proteins, VEGF family (angiogenesis), GLUT 1-3 (metabolic switch), etc. (Table 2). Mapping several distinct hypoxic mRNAs would provide hints of spatial indication of hypoxia-driven barriers in the different tumor areas, related to EMC remodeling, neo-angiogenesis, metabolic reprogramming, stem cell maintenance, etc. (physical or functional barriers).

**Table 2:** Set of candidate proteins to monitor transcriptionally, which are involved in different hypoxia-dependent phenotypes, leading to mechanical and functional barriers

Hypoxic-dependent phenotype	Proteins to monitor (mRNA)
ECM remodeling	ZEB1, ZEB2, MMP2, MMP9, bFGF, IL-6, PDGF, TGF-B, P4AH1, P4AH2, PLOD1, PLOD2, LOX
Metabolism switch	CAIX-XII, LDHA, MCT1, MCT4, SLC2A1, PDK1, ALDA, GLUT 1-3, PKM2
Angiogenesis	VEGFA, SCF, ANGPT2, SDF1, PDGFB, PGF, CTGF, FGF
Stem cell maintenance	ALKBH5, SIAH1, WWTR1, GCLM, BMP, JAK-STAT, SHH, SOX2, Oct-4, NANOG
Immune evasion	PDL1, CD39, CD47, CD73, CXCL12, ET-1, ET-2, SDF1, Sema3A

It could be of interest to collect spatial transcriptional data from different types of tumors at different stages and to create topographies of mRNAs organization for hypoxia and immune signatures. Obtaining correlations between markers of immune excluded areas and hypoxia-driven or hypoxia-independent barriers, would allow to investigate if one/multiple pathways creating physical or functional barriers are prevalent in human cancer at different stages, or these pathways just overlap randomly.

**Fig. 5:**
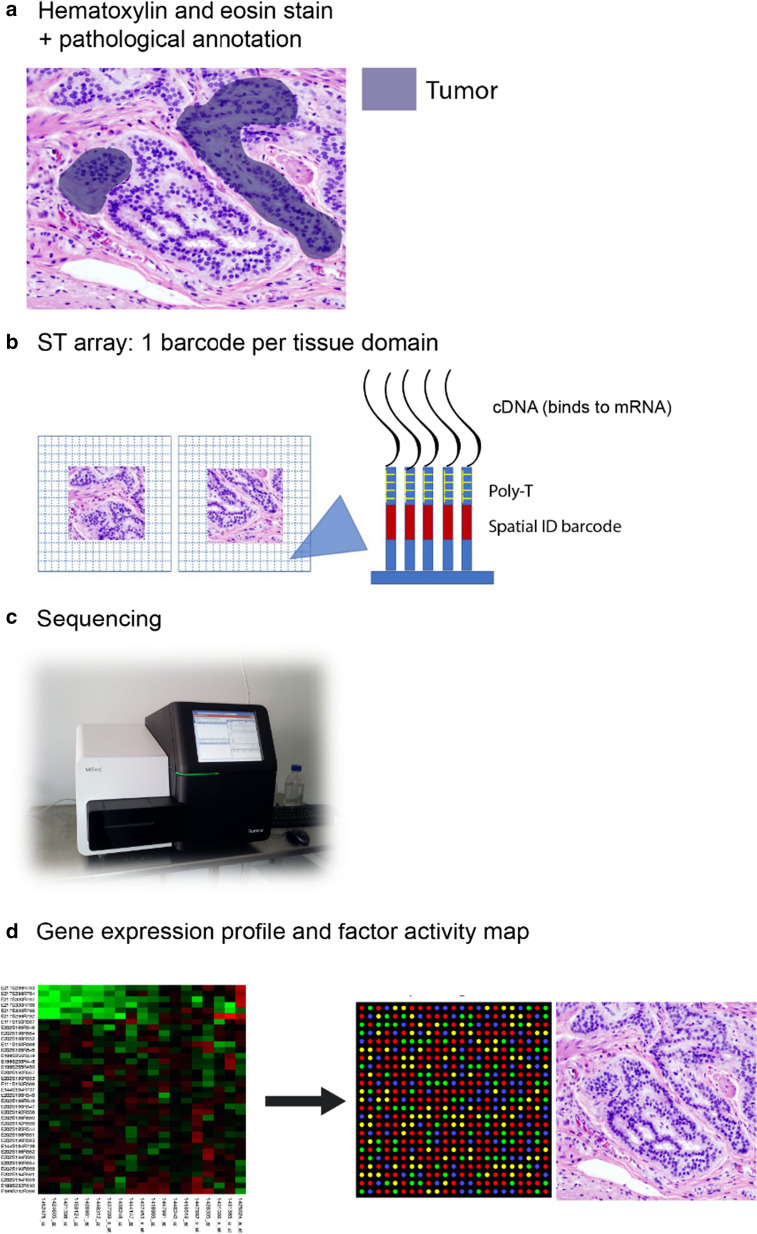
Spatial transcriptomics workflow including the downstream analysis. **a** Histological tumor sections are stained with hematoxylin and eosin-stained and imaged before permeabilization. **b** The sections are placed on glass slides containing RT-primers arrayed as spots that corresponded to tissues domains. The barcoded microarrays contain printed spots of RT-primers with unique barcode sequences. The RT-primers at each spot have a unique spatial ID barcode, which is sequenced along with the transcript to enable trace-back to a specific tissue domain. **c** Sequencing. **d** After sequencing the gene expression profiles and factor activity map are created

New spatial transcriptomic (ST) technologies take advantage of spatially barcoded oligo-deoxythymidine microarrays, allowing for unbiased mapping of transcripts (Fig. 5). Each area resolves the transcriptome of 10-200 cells depending on the tissue context [311]. ST has been used to study breast cancer, melanoma, prostate cancer, adult human hearth tissue, pancreatic ductal adenocarcinoma, mouse, human and mouse spinal cord tissue and mouse olfactory bulb [312–319]. Moncada et al., (2020) combined microarray-based ST that reveals spatial patterns of gene expression, with single cell RNA sequencing (scRNA-Seq) generated from the same sample, to identify enrichments of specific cell types and subpopulations across spatially-defined regions of pancreatic tumors [317]. Mohenska et al., (2019) combined ST with 3D modelling to investigate and map three-dimensionally the transcriptome in the mouse adult heart [319]. Philippeos et al. (2018) used a combination of comparative ST profiling of human and mouse dermis and scRNA-Seq profiling of human dermal fibroblasts, to define markers in fibroblast populations of the adult human skin [320].

A final example is the study performed by Thrane et al., (2018), who applied the ST technology to melanoma lymph node biopsies and successfully sequenced the transcriptomes of over 2200 tissue domains revealing a detailed landscape of melanoma metastases [315]. To our knowledge, ST has never been used to analyze the correlation of hypoxic-dependent and independent barriers with immune signatures.

Immunohistochemistry (IHC) can also be used to evaluate the concomitant expression of HIF-induced proteins and the infiltration of immune cells, in tumors [29, 321]. Exogenously administered nitroimidazole-based components accumulate in hypoxic cells and they can be detected by IHC, PET or SPECT imaging. A commonly used nitroimidazole molecule is called pimonidazole (Hypoxyprobe^TM^) and it has been shown that, immunochemical detection of pimonidazole protein adducts, can be successfully used to investigate hypoxia gradients in histological sections from tumors [322–325]. When oxygen level in tissues decreases below 1.3%, pimonidazole binds to thiol-containing proteins, peptides and amino acids, forming covalent bonds [326–328]. Hypoxic cells are detected using a specific monoclonal antibody and the amount of pimonidazole is directly proportional to the level of hypoxia in the tissue [322]. No toxic effects are associate with pimonidazole administration; therefore, this drug has also been used in patients. Pimonidazole administration followed by biopsy was performed to assess tumor hypoxia in cervical carcinoma, head and neck carcinoma and prostate cancer [323–325, 329, 330].

A pentafluorinated derivative of the 2-nitroimidazole etanidazole, called EF5, is also commonly used in IHC to detect gradients of hypoxia. EF5 is not toxic at doses adequate to detect tumor hypoxia and it is administered intravenously to patients. Tumors are biopsied approximately 48 hours after drug administration [331]. EF5 has been used on patients with squamous cell carcinoma: head and neck tumor, and uterine cervix cancer [332–334]. It has also been used on xenograft models of human colorectal carcinoma and sarcoma and in the detection of hypoxia in atherosclerotic plaques in mice [334–336].

Endogenous proteins such as HIF-1α can be tested by IHC, together with HIF-induced proteins. Such commonly used protein markers for hypoxia include CAIX, VEGFA, osteopontin, glucose transporter GLUT-1 and GLUT-3, BNIP3, PDK1, LDHA, LOX and EPO [321, 333, 337–339].

Regarding immune cells, the density of CD3, CD4, CD8, CD20, CD68, CD163, PD1 and FOXP3-expressing T lymphocytes is often used to evaluate immune infiltration and quantify it by digital pathology. In a recent publication, Kather et al., (2018) measured the topography of multiple immune cell types in a pan-cancer cohort by IHC, providing the first systematic analysis of hot, cold and immune excluded patterns and investigating how these patterns differ between immune cell types and cancer types [29]. Combining immunostaining of hypoxia-induced proteins and T cells in a similar pan-cancer cohort would provide correlations about pattern of immune exclusion and hypoxic related phenotypes.

Few disadvantages are associated with these approaches. Firstly, they require biopsies that precludes the serial monitoring of the same tissue at different time points. Second, the proper handling of samples is essential. For example, upon sample exposure to 20% oxygen, both HIF-1α and HIF-1β mRNA decrease to normoxic levels in less than 5 minutes. The half-life of the HIF-1α protein in normoxic conditions is also approximately 5 minutes [74, 76, 340, 341]. The stability of mRNA and protein levels for other hypoxic markers, such as VEGFA and CAIX is higher [342, 343]. Therefore, it is important to select the most appropriate markers according to sample processing procedure.

*In vivo* studies, correlating hypoxia and immune excluded phenotypes could be important for clinical monitoring (Fig. 4b). A robust technique could also be exploited as a predictive marker for immunotherapy efficiency and as a tool to monitor progression during treatment.

The methods described below provide a spatial characterization of hypoxic regions and allow the repetitive measurement of the same area at different time points. They provide measurements at various locations, allowing stratification of the information. Since different techniques document oxygen concentration in different locations (i.e. intracellular hypoxia, interstitial hypoxia or blood oxygenation), it is not possible to compare the values obtained with different methods, even within the same tissue.

Polarographic electrodes represent the gold standard for tumor hypoxia characterization in vivo [344, 345]. These invasive probes measure interstitial oxygen in sub-millimeter gradients, sampling a tissue volume of about 50-100 cells [346]. The probes are inserted into a tumor or metastatic lymph node. Variability in reported values could be due to the localization of the probes [347].

Ideally, minimally invasive screening techniques are generally highly preferred, especially for translational and clinical trials. Non-invasive imaging techniques include phosphorescence quenching, positron emission tomography (PET), blood oxygen level dependent (BOLD) magnetic resonance imaging (MRI), tissue oxygen level dependent (TOLD) MRI, oxygen-enhanced MRI (OE-MRI), near-infrared spectroscopy/tomography and single photon emission computed tomography (SPECT).

Phosphorescence quenching depends on the interaction of phosphorescent dyes with oxygen molecules. It is a direct measurement of the partial oxygen pressure and, unlike other methods, the reported value is independent of the tracer concentration [348, 349]. Molecular reporters are largely based on platinum or palladium containing porphyrins and they allow the imaging of tumors at specific absorbance/emission wavelengths [350]. Their emission decays exponentially, at a rate proportional to the local oxygen concentration therefore, increasing the oxygen pressure shortens the lifetime of the reporter, decreasing the total phosphorescence intensity [348, 351]. Due to technical challenges, this technique has not yet been used in clinical settings.

PET is a technique commonly used in the medical field. During recent years it has been shown to allow accurate diagnoses and increase patient comfort. PET allows the absolute quantification of intracellular hypoxia, spatially and temporally [352]. This methodology delivers information about inter- and intra-tumoral heterogeneity, in addition to the molecular and functional properties of a tumor. Small tracer molecules susceptible to hypoxic conditions are required and success of imaging is dependent upon their delivery. Properties of an ideal tracer include hypoxia-specificity, high stability, and effectiveness in clinical settings within a broad range of tumor cell types and volumes. The probe should have pharmacokinetic properties that are independent from parameters known to co-vary with hypoxia (i.e. pH, blood flow, etc.) and should be sufficiently lipophilic in order to enter cells. However, it should also be sufficiently hydrophilic to avoid membrane sequestration and have a faster clearance from the systemic circulation, normoxic and necrotic tissue [353, 354]. Small molecules containing 2-nitroimidazole undergo single electron reduction to form reactive radicals, accumulating under hypoxic conditions. They constitute a versatile class of probes, which can be conjugated with a radioisotope for imaging; over the past years several derivatives have been developed [350, 353].

**Fig. 6:**
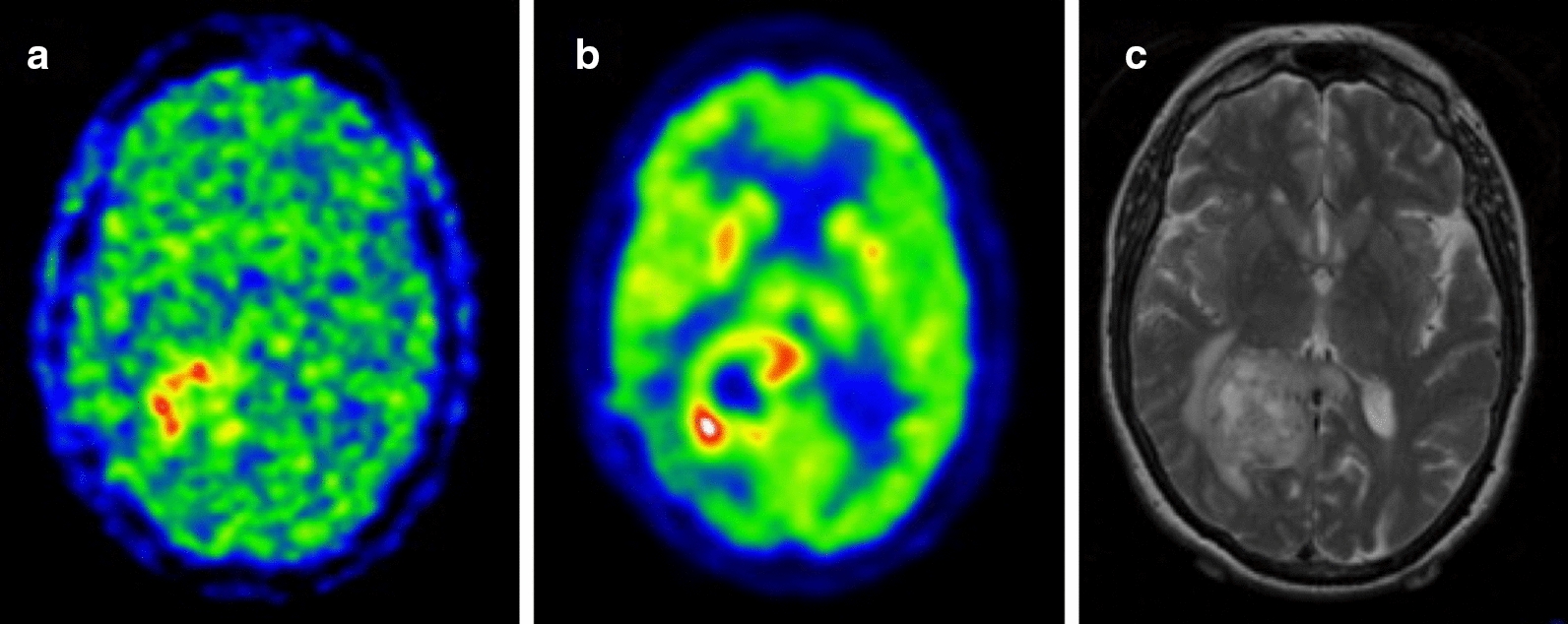
Examples of hypoxia imaging in glioma. Postsurgical assessment of residual tumor showing **a** 18F-FMISO gradient, hypoxic areas are found in the surgical margin of the tumor. **b** 18F-FDG staining, marker for the tissue uptake of glucose and C) preoperative MRI showing the tumor mass in the right posterior parieto-occipital lobe

Widely used 2-nitroimidazole derivatives are 18F-labeled tracers (MISO, FMISO, FETNIM, FRP170, EF5, EF3, FAZA and HX4). None of these probes bear all the characteristics mentioned above therefore, there is currently no optimal and standardized quantification methodology for imaging hypoxia in all cancer types. Moreover, the tracers have different properties, and as such, the comparison of studies with different tracers is not recommended. 18F-FMISO enters cells via passive diffusion, where it is reduced by nitroreductase enzymes under hypoxic conditions. It forms reactive oxygen species, then binds to macromolecular cellular components and conjugates with glutathione, which causes its confinement in the cellular compartment [355–357]. 18F-FMISO is the most widely used tracer but is found at a relatively low concentration in tumors. Moreover, it is normally imaged several hours post-injection, due to its slow clearance from background tissues [358–360]. These circumstances could render the image acquisition challenging. The 18F-FMISO has been used to detect hypoxia in patients with gliomas, head-and-neck, breast, lung, pancreatic and renal tumours [352, 361–374] (Fig. 6). However, 18F-FMISO retention in sarcomas is variable [375–377].

18F- FETNIM, FRP170, EF5, EF3, FAZA and HX4 are next-generation more hydrophilic hypoxia probes and they are dependent on renal clearance. Although these tracers improve the resolution of hypoxia-to-normoxia tissue ratios (higher contrast) compared to 18F-FMISO, intrinsic differences in kidney clearance could lead to variability in measurements [378–381]. 18F-FAZA and other 18F-labelled tracers were used to detect gradients of hypoxia in patients with lung cancer, gliomas, lymphomas, prostate, pancreatic, cervical, rectal and head-and-neck and rectal tumours [382–397].

The lack of a standardized method renders crucial the assessment of the most suitable hypoxia tracer case by case, depending upon the physiological and pathological conditions. Moreover, discrepancies in reproducibility among studies could be due to differences in the methodological parameters (image acquisition protocol, selection of hypoxic-normoxic thresholds) and to the relatively small number of patients tested [353].

Cu-ATSM is an alternative class of agents used in the study of hypoxia with PET. It is characterised by a low molecular weight and lipophilicity, resulting in high membrane permeability. Cu-ATSM is based on a complex of copper with diacetyl-bis (N4-methylthiosemicarbazone) ligands and its specificity is due to the intracellular reduction of Cu(II) to Cu(I) under hypoxic conditions. The complex Cu(I)–ATSM is unstable and may further dissociate into Cu(I) and ATSM, leading to the intracellular trapping of the Cu(I) ion [353, 398].

Cu-ATSM was successfully used in clinical settings to monitor hypoxia in cervical, head and neck, lung and rectal cancer patients [399–404].

SPECT also requires a tracer which reports on intracellular hypoxia and allows the quantification of data at a macroscopic scale, in tumor regions [353]. SPECT is more commonly used in clinical settings than PET as the cost of tracer Technetium-99m is lower, it has a convenient half-life and versatile chemistry compared to 18F compounds [405]. However, PET has a superior spatial resolution and is considered a more suitable and accurate method for detecting tumoral hypoxia [406–408].

Magnetic resonance imaging may provide a practical and safer translational alternative to PET. BOLD-MRI is the standard methodology used to obtain images in functional MRI. It monitors blood oxygen levels using deoxy-haemoglobin as an endogenous marker, but does not provide a direct measurement of the oxygen tension in tissues. It is used to examine the variation in tissue oxygenation rather than quantitatively mapping hypoxia [350, 409–411]. Using this technique, Baudelet et al. (2006) observed signal fluctuations more frequently in tumor regions containing immature blood vessels in fibrosarcoma-bearing mice [412]. BOLD-MRI was used in clinical settings to evaluate response to treatments in recurrent cervical cancer and locally advanced breast cancer [413–415]. It was also used to generate oxygen saturation maps in normal and cancer prostate tissues and in patients with infiltrative astrocytoma [415–417].

**Fig. 7:**
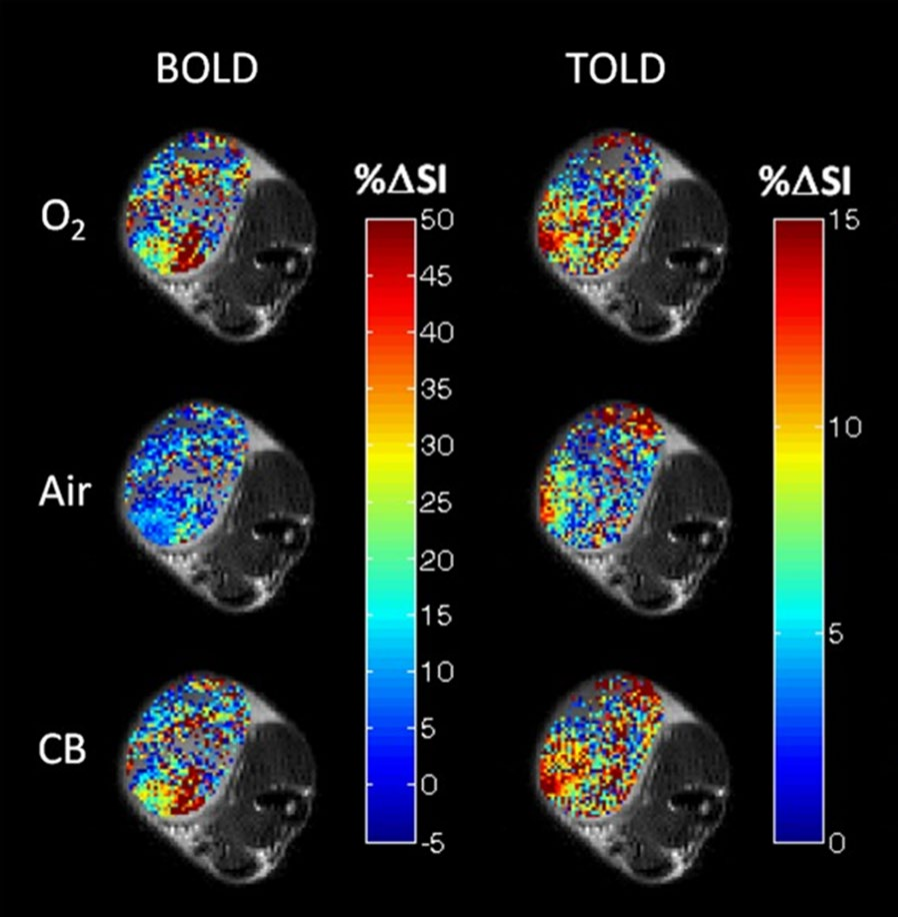
Comparative study of BOLD and TOLD to assess tumor oxygenation. BOLD and TOLD-MRI measures of oxygen gradients overlaid on a high-resolution image of a small Dunning prostate R3327-AT1 tumor in conditions of hyperoxia, 100% O_2_ (O_2_), return to normal air (Air) and carbogen breathing, 95% O_2_ and 5% CO_2_ (CB). Changes in signal intensity is represented by %ΔSI. Response maps compare final images versus baseline. Each map shows substantial intratumoral heterogeneity and differences in the heatmap pattern within the same treatment, between BOLD and TOLD

TOLD-MRI offers more robust insights into tumor hypoxia. It measures the concentration of free oxygen molecules in the plasma and interstitial tissue fluid. TOLD-MRI provides values on the partial oxygen pressure in tissues. Moreover, it does not require the use of contrast agent, making it more cost-effective than PET [418]. Preclinical studies have shown that TOLD-MRI is a predictor of radiotherapy outcomes and a potential prognostic biomarker (Fig. 7). TOLD-MRI has also been used to confirm tumor re-oxygenation after treatment, in patients affected by glioblastoma [419–421].

Another technique that provides evidence about tumor oximetry, together with microvascular permeability, is the oxygen-enhanced MRI. OE-MRI uses a low-field magnetic resonance scanner and paramagnetic contrast agent allowing spatial resolution of ≈1 mm and temporal resolution of 2 minutes [422]. OE-MRI does not provide quantitative measures of oxygen per se but maps oxygen delivery in tissues with fully saturated haemoglobin [423, 424]. Its potential was assessed in preclinical studies, in healthy individuals subjected to respiratory challenges and in patients with lung cancer and advanced cancer of the abdomen and pelvis [425–427].

There is currently no standardized non-invasive technique that is accurate for all tumor types and available for routine clinical use. Therefore, depending on the pathophysiological features to be investigated, it is important to evaluate the correct methodology to adopt.

Significant advancements were achieved in the last few years in imaging immune cells in the context of translational research [428, 429]. Tracking T cells in vivo implies the need for non-invasive, highly sensitive, molecular quantitative and total body techniques such as PET, SPECT and MRI. The development of the immuno-PET allows to monitor different types of immune cells [430–433]. To be detected, T cells are provided with a tracer conjugated to a molecular target. Example of targets are molecules on the T-cell surface (TCR complex), metabolic pathways, T-cell activation markers (PD-1, OX40, IL-2), PET reporter genes and T-cell effector function markers [429].

Coupling in vivo detection of hypoxia gradients and T cell distribution could provide crucial information to predict immunotherapy efficiency. Such analysis could also provide some insight into the spatial and dynamic organization of determinants responsible for phenomenon of immune exclusion.

## Conclusions

Immunotherapy has to overcome various challenges in order to increase its therapeutic potential in solid tumors. Despite evidence that hypoxia is a crucial determinant for the efficacy of immunotherapies, few clinical trials are studying how these two aspects are related in patients.

Preclinical research demonstrated the existence of a plethora of factors and receptors shaping the hypoxic phenotype. Moreover, the dynamics of these factors change during time and, likely, they also vary according to tumor type.

However, it is unclear if hypoxia could be considered an upstream determinant triggering the immune excluded phenotype. Further studies are required to understand the spatial and temporal evolution of mechanisms involved in the immune exclusion phenomenon. It would also be of interest to correlate in vivo tumor stages with immune infiltration and hypoxic-related markers, in a pan-cancer investigation. This correlation would provide insights into strategies to adopt in order to increase immunotherapy efficiency and rationale for translational combinatorial treatments. Moreover, together with ex vivo techniques it could be possible to integrate the actual knowledge of distinct mechanisms responsible for the immune excluded phenotype, into a unified ‘*Theory of Everything*’.

## Data Availability

Not applicable

## Abbreviations

CAR-T: Chimeric antigen receptor T cells
TIL: Tumor infiltrating lymphocytes
SITC: Society for Immunotherapy of Cancer
ICR: Immunologic constant of rejection
TIS: Tumor inflammation signature
HREs: Hypoxia regulated elements
HIFs: Hypoxic inducible factors
ODD: Oxygen degradation domain
PHDs: Prolyl hydroxylases
pVHL: von-Hippel Lindau tumor suppressor
PI3K: Phosphoinositol 3-kinase
MAPK: Mitogen-activated protein kinase
mTOR: Mammalian target of rapamycin
ROS: Reactive oxygen species
TME: Tumor microenvironment
ECM: Extra-cellular matrix
Ang-2: Angiopoietin-2
TGF-β: Transforming growth factor beta
PDGFB: Platelet-derived growth factor B
PGF: Placental growth factor
CTGF: Connective tissue growth factor
SCF: Stem cell factor
CXCL12: Stromal cell-derived factor 1
ET-1: Endothelin-1
AP-1: Binding of activator protein-1
EGF: Epidermal growth factor
PDGF: Platelet-derived growth factor
FGF2: Fibroblast growth factor 2
CAFs: Cancer-associated fibroblasts
PDA: Pancreatic ductal adenocarcinoma
EMT: Epithelial–mesenchymal transition
TNF-α: Tumor necrosis factor α
IL: Interleukin
HGF: Hepatocyte growth factor
bFGF: Basic fibroblast growth factor
EGF: Epidermal growth factor
ZEB: Zinc finger E-box binding homeobox
MMPs: Matrix metalloproteinases
FLG: Filaggrin
GLUTs: Enzymes glucose transporters
PDK1: Pyruvate dehydrogenase kinase 1
LDHA: Lactic dehydrogenase A
PKM2: Pyruvate kinase M2 subtype
MCTs: Mono-carboxylate transporters
CAs: Carbonic anhydrases
GLS1: Glutaminase 1
T regs: T regulatory cells
TAMs: Tumor-associated macrophages
MDSCs: Myeloid-derived suppressor cells
MHC-I: Major histocompatibility class-I
JAK: Janus kinase
STAT: Transducer and activator of transcription
CSCs: Cancer stem cells
ST: Spatial transcriptomic
scRNA-Seq: Single cell RNA sequencing
IHC: Immunohistochemistry
PET: Positron emission tomography
BOLD: Blood oxygen level dependent
MRI: Magnetic resonance imaging
TOLD: Tissue oxygen level dependent
OE: Oxygen-enhanced
SPECT: Single photon emission computed tomography
SCNA: Somatic copy number alteration
